# General practitioners can increase participation in cervical cancer screening – a model program in Hungary

**DOI:** 10.1186/s12875-018-0755-0

**Published:** 2018-05-19

**Authors:** Anikó Gyulai, Attila Nagy, Vera Pataki, Dóra Tonté, Róza Ádány, Zoltán Vokó

**Affiliations:** 1 Department of Public Health, Faculty of Health, University of Debrecen, Sóstói u. 2-4, Nyíregyháza, H-4400 Hungary; 2 Department of Preventive Medicine, Faculty of Public Health, University of Debrecen, Kassai út 26, Debrecen, H-4028 Hungary; 3 GlaxoSmithKline Hungary Ltd., Csörsz u. 43, Budapest, H-1124 Hungary; 4 Department of Health Policy & Health Economics, Institute of Economics, Faculty of Social Sciences, Eötvös Loránd University, Pázmány Péter sétány 1/a, Budapest, H-1117 Hungary

**Keywords:** Cervical cancer screening, General practitioner, Health communication, Health behavior, Hungary

## Abstract

**Background:**

Cervical cancer is a preventable disease. Unfortunately, its mortality is high in Hungary: 9.2 deaths /100000 women/year in 2015. The Hungarian organized, nationwide cervical screening program was launched in 2003, but it could improve the coverage rate of cervical cancer screening only by a few percentage points. The vast majority of women still uses opportunistic screening and the organized screening program had little impact on participation by women who never or rarely consult their gynecologists. We assessed whether involving general practitioners in the cervical cancer screening process would increase participation.

**Methods:**

The study consisted of two parts: 1. A questionnaire-based health survey was conducted using a representative sample of women aged 25 to 65 years from 11 Hungarian counties, in which we studied where women obtained information about cervical cancer screening. 2. Additionally, a model program and its evaluation were implemented in the practices of general practitioners in one of the 11 counties (Zala county). In this program, general practitioners were informed of their patients’ participation in the cervical cancer screening program, and they motivated those who refused the invitation.

**Results:**

Questionnaire-based health survey: A total of 74% (95% confidence interval (CI): 70–77%) of the target population had a screening examination within the previous 3 years. The majority (58, 95% CI: 54–62%) of the target population did not ask for information about cervical cancer screening at all. Only 21% (95% CI: 17–26%) consulted their general practitioners about cancer screening. Evaluation of the model program: the general practitioners effectively motivated 24 out of 88 women (27, 95% CI: 18–38%) who initially refused to participate in the screening program.

**Conclusion:**

The majority of Hungarian women are not informed about cervical cancer screening beyond the invitation letter. General practitioners could play a more important role in mobilizing the population to utilize preventive services. The involvement of general practitioners in the organization of the cervical cancer screening program could increase the participation of those women who generally refuse the services.

**Electronic supplementary material:**

The online version of this article (10.1186/s12875-018-0755-0) contains supplementary material, which is available to authorized users.

## Background

**Fig. 1:**
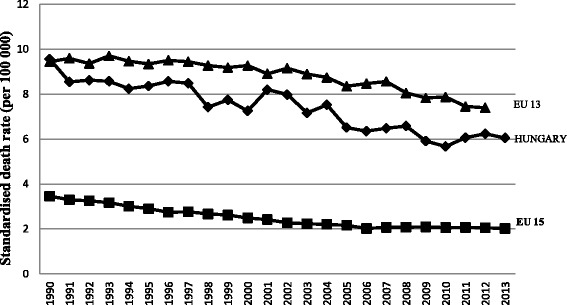
Cervical cancer mortality in Hungary and in EU countries from 1990 to 2013. EU-15: countries that joined the European Union before 2004; EU-13: countries that have joined the European Union since 2004. Source: European Health For All Database. World Health Organization Regional Office for Europe, Updated January 2016. Standard population: European Old Standard Population

The mortality rate for cervical cancer is very high in Hungary compared with the average in western European countries (Fig. 1) [1]. More than 400 women die of this disease each year in a country with a total female population of 5.28 million [2]. Opportunistic complex gynecological screening (including colposcopic screenings) and cytological examination with Papanicolaou smears have a long tradition in Hungary [3]. However, this tradition has not translated into a decrease in cervical cancer mortality rates. Therefore, national-level call-and-recall-based organized cervical cancer screening began in 2003 in Hungary [4]. Women between 25 and 65 years of age are called for a screening every 3 years by the National Public Health Service [5, 6]. Traditionally, the great majority of eligible women visit their gynecologists, at least within every 3 year, who perform a complete gynecological examination along with smear-taking for cytological examination. In this way, a large number of cervical screening examinations take place outside organized screening settings [7]. Unfortunately, the national cervical cancer screening program increased the proportion of those who received a screening by only a few percentage points [8, 9]. In 2011 Eurostat reported that cervical cancer screening participation rate was low in Hungary. The percentage of women who underwent at least 1 Pap test in a 3-year period was 35.94%. [10]. Participation in a screening program is known to be closely related to socioeconomic factors, cultural factors and attitudes [11, 12]. According to surveys, the majority of women not attending screenings have low socioeconomic status, and see a gynecologist only if they have serious complaints. Other reasons are lack of time and ignorance of the importance of screening [13].

As general practitioners provide health service closest to the population, they are respected, trustworthy members of the local community and can have a key role in health communication, including mobilization of the population to participate in screening programs [14].

### The aim of the study

The aim of our study was to assess where women obtain information about cervical cancer screening and to test whether the involvement of general practitioners in the cervical cancer screening program process could increase the participation rate.

## Methods

### Studies performed

This study was performed within the framework of the General Practitioners’ Morbidity Sentinel Station Program (GPMSSP). The GPMSSP is the first representative chronic disease morbidity monitoring program in Hungary [15]. More than 200 general practitioners from 11 counties report the occurrence of non-communicable diseases with major public health importance via a quality managed system. In addition to continuous monitoring, the program provides a research framework for epidemiological and health services research.

This study was conducted in two parts:

1. A questionnaire-based health survey in a representative group of 25- to 54-year-old women belonging to the practices of general practitioners participating in the GPMSSP
2. Screening model program and its evaluation: involvement of general practitioners in the cervical cancer screening program process in Zala county.

### Participants

Out of the 200 GPs who participated in GPMSSP 96 volunteered to participate in this study. In these practices 59,730 women aged 25–65 years were registered. In the first step of selecting the study population we defined the necessary sample size by county on the basis of the size of the female population aged 25–65 years in the target population (Baranya, Bács-Kiskun, Borsod-Abaúj-Zemplén, Győr-Moson-Sopron, Hajdú-Bihar, Heves, Jász-Nagykun-Szolnok, Komárom-Esztergom, Nógrád, Szabolcs-Szatmár-Bereg and Zala). In the second stage we set the necessary sample size for each general practice taking into account the number of women aged 25–65 years registered in the GP practices participating in the GPMSSP. The final size of the random sample was 1306 persons. Women living in Zala county were not included in this process.

In the power calculations of the evaluation of the model program the minimum detectable success rate was 20% with ±10% uncertainty. We planned the sample size in such a way, that if indeed this minimum effect is achieved then the lower boundary of the 95% confidence interval of the effect estimate would not include 10%. We assumed at least 45% participation in the survey, 40% participation rate in the intervention study, 80% coverage of the cytological test among the participants of the survey in Zala county who would consent to participate in the intervention pilot. This resulted in a necessary sample size of 1806 in Zala county. To be on the safe size, 2000 persons were involved in the survey in Zala county. The sub-sample of the intervention model program in Zala county was selected from women aged 25–65 years registered at the practices of the GPs participating in the model program.

**Fig. 2:**
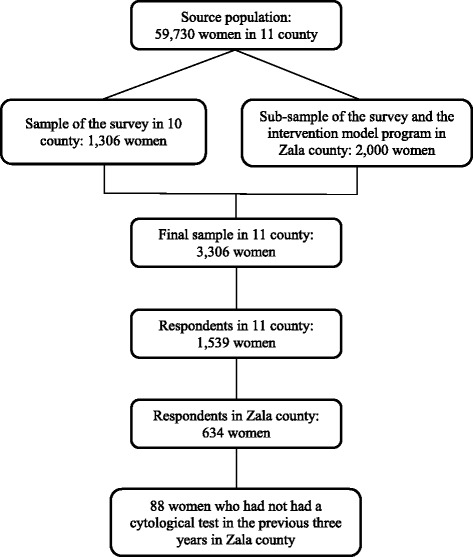
Selection process of the sample for the intervention in Zala County

Altogether 3306 women were selected as the final sample for the program. (Fig. 2.) This oversampling was taken into account in the analysis of the survey by using appropriate weights.

### Data collection

A questionnaire containing questions regarding demographic and socioeconomic factors, health status, lifestyle (including physical activity, diet, smoking and sexual activity), knowledge about cervical cancer, utilization of gynecological services, participation in the cervical cancer screening program, and the source and content of information obtained about the screening program was developed [Additional file 1]. The questionnaire was tested in focus groups and was further developed before application in our study. It took approximately 25 min to complete the questionnaire. The questionnaires, along with letters containing instructions, were sent to all participants through their general practitioners to be answered anonymously. Participants were asked to send back the questionnaire by mail. We have previously reported the determinants of participation in a screening program and the major causes of refusal [13]. In Zala county, women were additionally asked to provide written consent to participation in the model program. If they consented, then they also agreed that their personal data could be used to contact them, to track their data in the registries of the cytology labs in the county, and to inform their general practitioners about their participation in the cervical cancer screening program.

### Intervention model program

We selected all participating women from Zala county who had not taken part in cervical cancer screening within 3 years prior to the survey but were willing to participate in the intervention model program (Fig. 2.). Their general practitioners were informed, and the general practitioners, in collaboration with the county screening organizing officer, sent them an invitation to the screening together with an information leaflet. The women’s participation in the screening program was monitored by the registries of the cytological laboratories in the county, which all agreed to participate in the study.

During the implementation of the screening program each general practitioner acted according to the study protocol by following these steps:

- General practitioners sent information letters to women to be involved in this part of the study with an invitation to screening and enclosed a leaflet about some useful information how to prevent cervical cancer. Each of the 88 women received the same letter but each information letter was signed by the respective general practitioner.
- After that, the general practitioners got reports weekly about who had taken part in the screening from the coordinator of Zala county, who received this information from the cytological labs.
- Six weeks after sending the letters, the general practitioners tried to reach those women who had not taken part in the screening but at that time in person or by phone. The general practitioner tried to motivate and persuade them to cooperate and to participate in the screening program.
- The participation in screening was observed for a further 4 weeks based on the reports of the cytological labs. The screening model program was terminated 4 weeks after the last call or personal invitation.

### Statistical analysis

We estimated the frequencies of different characteristics in the target population by weighting for the age distribution of the counties to correct for refusal. The survey-analysis module of the statistical package STATA was used for the analysis [16].

## Results

### Survey about obtaining information about the cervical cancer screening program in the 11 counties

Of the 3306 questionnaires that were sent, 1539 (47%) were received by the research center. The study participants were women between the ages of 25 and 65 years and were 45.6 years old on average (standard deviation [SD] 11.4). A total of 76% lived with a partner, 21% had only a primary school education, and 18% had completed higher education. Table 1 shows the demographic characteristics of the target population, based on the weighted analysis (Table 1). Of the target population, 74% (95% confidence interval (CI) 70–77%) had a cytological examination within the previous 3 years, within or outside the organized screening program. Only 35.3% (95% CI: 32.9–37.7) of the 25- to 65-year-old women from 11 counties were invited to the organized cervical cancer screening, i.e., only those who did not have an opportunistic screening test, and only half of those (53.6, 95% CI: 49.4–57.8%) participated. A total of 58% (95% CI: 54–62%) of the target population did not ask for any information about cervical cancer screenings. Among those who asked for information about screenings, the majority obtained information from a gynecologist (65, 95% CI: 59–71%) and one-third from the media and leaflets provided by the health service. General practitioners were mentioned as a source of information by only 21% (95% CI: 17–26%) of women (Table 2). Interested women primarily asked whether screening was reliable and capable of detecting early changes (44, 95% CI: 38–50%) and asked when the screening results would be available (44, 95% CI: 38–50) (Table 3).

**Table 1:** Demographic characteristics of the target population

	Proportion (%)	95% Confidence interval (CI)
Age (years)		
25–34	26	23–30
35–44	23	20–26
45–54	27	24–30
55–65	24	21–27
Education		
primary education	21	18–24
secondary education without final examination	21	18–25
secondary education with final examination	30	26–33
post-secondary education without diploma	10	8–13
college or university degree	18	15–21

**Table 2:** Proportion of women obtaining information about cervical cancer screening from different sources

	Proportion^a^ (%)	95% Confidence interval (CI)
Gynecologist	65	59–71
TV, radio	36	30–42
Leaflet found in the consultation-room	30	25–36
Newspaper	24	19–29
Friends, colleagues	24	19–29
Internet	22	17–27
General practitioner	21	17–26
Family members	20	16–25
Specialized textbooks	19	14–24
District nurse	14	10–19
Oncologist	6	4–10
Non-governmental organization	1	0.3–3
Pharmacist	0.5	0.1–1.5
Human papilloma virus outpatient service settings	0.3	0–2

^a^More answers were available for selection

**Table 3:** The type of information women obtained about cervical cancer screening

	Answers^a^ mentioned (%)	95% Confidence interval (CI)
Can screening reveal all early changes?	44	38–50
When do I receive the result of the screening?	44	38–50
How often do I have to attend the screening?	35	30–41
What happens if the result is unfavorable?	32	27–37
What does an unfavorable result mean?	28	22–33
How and who will notify me?	27	22–32
Where can I undergo screening?	25	20–30
Where does the screening test take place, and what does the doctor do?	25	20–30
Is screening pain-free?	25	20–30
From which age is screening recommended?	23	19–29
Who performs the screening?	17	13–21
How long does the screening test last?	9	6–12
Is screening risky, and can it cause any damage?	7	4–10

^a^More answers were available for selection

### Intervention model program and its evaluation in Zala county

We selected 88 of the 634 women who had agreed to take part in the screening model program in Zala county and who had not participated in a cervical cancer screening in the previous 3 years. Women who did not participate in the screening program was older, had lower incomes and were less educated than women who participated in it in Zala county. (Table 4.)

**Table 4:** Socio-demographic characteristics of the study population from Zala county according to participation in cervical screening within three years

Characteristics	Participating in screening (*n* = 546)	Non-participating in screening (*n* = 88)	*p*-value
Age	43.87 (11.88)	51.44 (11.17)	^*^p<0.001
Household equivalent monthly income (Euro)	442.32 (188.83)	386.82 (170.75)	^*^p = 0.021
Employed	69.9	46.4	^**^p<0.001
Education			^**^p = 0.006
primary education	8.3	19.8	
secondary education without final examination	23.1	24.4	
secondary education with final examination	30.3	25.6	
post-secondary education without diploma	11.4	14.0	
college or university degree	27.0	16.3	

Numbers are mean (SD) or percentages. * independent samples t-test; ** chi-square test

As a result of the intervention model program 24 of the 88 women (27, 95%CI: 18–38%) participated in the screening program. Of the successful cases, there were 17 occasions in which a letter from the general practitioner was sufficient and 7 instances in which a personal conversation changed the mind of the participant.

## Discussion

Cervical cancer is a preventable cancer; evidence of the success of cytological screening is indicated by the low incidence of invasive cervical cancer in well-screened populations and by the high incidence and mortality in populations without screening [17]. In 2013, the mortality rate was 6.02 per 100,000 women in Hungary. Based on the most recently available data, morbidity rates have declined over the past decades, with the mortality rate essentially remaining constant (5–6/100,000 women/year) since 2005 [1]. Although the organized screenings began in 2003, the number of sample tests performed outside the program is still 20 times higher than the number performed within the screening program [7, 18]. According to our survey, the overall screening rate in Hungary (74%) is not low. The real frequency, however, is likely to be lower because of selection bias associated with a relatively low response rate of 47%. It is reasonable to assume that, on average, the level of health consciousness was higher among the respondents than that among the target population [13]. GPs’ frequent contact with the public and their credibility offer a great potential for cancer prevention [19]. Effective communication is a key success factor for an organized screening program [20]. Our results showed that the majority of the target population did not receive any information about the screening program. Effective communication should be an inherent part of the screening program in order to enable the target population to make informed decisions about participation [20–22]. According to the results of our investigation, women asked for and received relevant information mainly from their gynecologists and characteristically not before screening but rather in the framework of the gynecological examination.

General practitioners had a very limited role in the cancer screening program in Hungary. The results obtained from the Zala county model project showed that the involvement of general practitioners in the cervical cancer screening program process could increase the participation of women who generally refuse these services. In the model program, a quarter of the women who typically did not take part in cervical cancer screening underwent the screening after advice from their general practitioner. Some recent developments in the Hungarian health care system have facilitated the participation of general practitioners in preventive activities. General practitioners gained online access to the records of their patients in the information system of the National Health Fund, and thus, they could obtain information about the type of outpatient and in-patient services their patients received, including screenings. Furthermore, the financing system for general practice, which still largely depends on capitation, was extended to performance-based financing [23, 24]. A small proportion of general practice fees currently depend on performance indicators. Participation of the target population in mammographic screening is already included in the list of indicators, but this is not yet the case for cervical cancer screening.

In developing a screening program, it is important to increase its accessibility, especially for low-income families and Romani people [25, 26]. Locally driven strategies that take into account the sociocultural beliefs and values of families can minimize barriers to cervical cancer prevention and control [27, 28]. If smears were taken in primary health care settings by GPs or nurses (as is standard practice in several Western European countries [29]), then participation rates in Hungary would probably increase. Primary health care services are more accessible to the general population. Staff are familiar with the cultural and socioeconomic background of the local population and could also be involved in local communication efforts regarding the program. It is important that the service utilization information is integrated with the screening information system. This is organized in Hungary in the framework of the cooperation between the National Health Insurance Fund and of the National Public Health Service. The recent development of the Hungarian e-health program, especially the establishment of the National Electronic Health Records, which centrally stores all medical records of every patient provides an opportunity for GPs themselves to follow-up their patients. To increase the accessibility of the cervical cancer screening program, the National Public Health Service has recently expanded the number of service providers. Health visitors were trained to take the smear and to provide the service locally, even in small villages. Smear taking has been integrated into the graduate training of health visitors, who traditionally provided only maternal and child care [30].

Pilot Health Visitor’s Cervical Screening Programs were introduced in 2009, and 15.8% (2009), 11.1% (2010) and 14.9% (2011) of the invited women appeared in each year for the request of the health visitors [31]. The Health Visitor’s Cervical Screening Program was later institutionalized, and currently it operates as part of the national public health service. Its effectiveness can further be enhanced by the involvement of the GPs in health communication and motivation of the target population. Developing a community-based intervention that is evidence based and theoretically grounded is challenging and time intensive [32]. It requires collaboration of different professionals, including medical and health professionals, psychologists, and sociologists.

Limitations of our study were the low response rate in the survey and the relatively small sample size in the intervention study. The main strength of our study was that it resulted in collaboration between primary care, the public health service and the cytology labs in one research project which served as a proof of concept to improve the screening program.

## Conclusion

Our results reaffirm that the capacity of primary health services could be more efficiently utilized in preventive care in Hungary.

Community-oriented health services need to be reorganized. A new framework of cooperation is needed from the National Public Health Services, screening coordinators, general practitioners, nurses, health visitors and patients.

## Additional file

Additional file 1 Questionnaire. (DOCX 44 kb)

## References

[1] WHO European Health For All Database. http://www.euro.who.int/en/what-we-do/data-and-evidence/databases/european-health-for-all-database-hfa-db2/offline-version. Accessed 20 Feb 2017.

[2] Demographic Yearbook 2012. Budapest (HU): Hungarian Central Statistical Office; 2013.

[3] Döbrőssy L (2007). Méhnyakrákszűrés 5 évtizede Magyarországon [Five decades of cervical cancer screening in Hungary]. Nőgyógyászati Onkológia.

[4] Kovács A, Döbrőssy L, Budai A, Boncz I, Cornides Á. A népegészségügyi méhnyakszűrés helyzete Magyarországon 2006-ban. [The state of organized cervical screening program in Hungary in 2006]. Orv Hetil. 2007;148(12):535–40. 10.1556/OH.2007.28075 17444018

[5] Országos Tisztifőorvosi Hivatal Méhnyakszűrési Munkacsoport: Lakossági méhnyakszűrés az „Egészség Évtizede” program keretében: törekvések a nőgyógyászati rákszűrés korszerűsítésére Magyarországon. [Working Group of Cervical Cancer Screening of National Chief Medical Officer’ Office: Mass screening of the uterine cervix in the program of the “Decade of Health”: perspectives of gynecologic cancer screening in Hungary.] Orv Hetil. 2004;145(1):35–40. [Hungarian] 15222140

[6] Boncz I, Sebestyén A, Ember I (2007). Organized, nationwide cervical cancer screening programme in Hungary. Gynecol Oncol.

[7] Kovács A, Döbrossy L, Budai A, Boncz I, Cornides A (2008). Cervical screening in Hungary: why does the “English model” work but the “Hungarian model” does not?. Eur J Gynaecol Oncol.

[8] Boncz I, Sebestyén A, Döbrőssy L, Kovács A, Budai A, Székely T (2007). A méhnyakszűrés részvételi mutatói Magyarországon [The coverage of cervical screening in Hungary]. Orv Hetil.

[9] Kovács A, Boncz I (2009). A szekunder prevenciós onkológiai szűrési programok helyzete Magyarországon. [the state of the organized oncological screening programs in Hungary]. Népegészségügy.

[10] Eurostat Database: Breast cancer and cervical cancer screenings [hlth_ps_scre] http://appsso.eurostat.ec.europa.eu/nui/show.do?dataset=hlth_ps_scre&lang=en Accessed 2 Jan 2018.

[11] Lofters AK, Schuler A, Slater M, Baxter NN, Persaud N, Pinto AD, et al. Using self-reported data on the social determinants of health in primary care to identify cancer screening disparities: opportunities and challenges BMC Family Practice. 2017; 10.1186/s12875-017-0599-z. 10.1186/s12875-017-0599-z PMC5330155 28241787

[12] Palència L, Espelt A, Rodríguez-Sanz M, Puigpinós R, Pons-Vigués M, Pasarín MI (2010). Socio-economic inequalities in breast and cervical cancer screening practices in Europe: influence of the type of screening program. Int J Epidemiol.

[13] Gyulai A, Nagy A, Pataki V, Tonté D, Ádány R, Vokó Z (2015). A survey of participation in an organised cervical cancer screening programme in Hungary. Cent Eur J Public Health.

[14] Austoker J (1994). Cancer prevention in primary care: screening for cervical cancer. BMJ.

[15] Széles G, Vokó Z, Jenei T, Kardos L, Pocsai Z, Bajtay A (2005). A preliminary evaluation of a health monitoring programme in Hungary. Eur J Pub Health.

[16] Stata Statistical Software Release 8.2 StataCorp LP, College Station, 2003; USA.

[17] Vesco KK, Whitlock EP, Eder M, Lin J, Burda BU, Senger CA (2011). Screening for cervical Cancer: a systematic evidence review for the U.S. preventive services task force. Evidence synthesis no. 86. AHRQ publication no. 11–05156- EF-1.

[18] Anttila A, Ronco G (2009). Working group on the registration and monitoring of cervical Cancer screening Programmes in the European Union; within the European network for information on Cancer (EUNICE). Description of the national situation of cervical cancer screening in the member states of the European Union. Eur J Cancer.

[19] McIlfatrick S, Keeney S, McKenna H, McCarley N, McElwee G. Investigating the role of the general practitioner in cancer prevention: a mixed methods study. BMC Fam Pract. 2013; 10.1186/1471-2296-14-58. 10.1186/1471-2296-14-58 PMC3653692 23651706

[20] Rimer BK, Briss PA, Zeller PK, Chan EC, Woolf SH (2004). Informed decision making: what is its role in cancer screening?. Cancer.

[21] Giordano L, Webster P, Anthony C, Szarewski A, Davies P, Arbyn M, Segnan N, Austoker J (2008). Improving the quality of communication in organised cervical cancer screening programmes. Patient Educ Couns.

[22] Villani J, Mortensen K (2013). Patient-provider communication and timely receipt of preventive services. Prev Med.

[23] Kolozsvári LR, Rurik I. A háziorvosok teljesítményének minőségi értékelése. Mi a probléma a háziorvosi indikátorokkal? [evaluation of the quality of performance of general practitioners. What is the problem with primary care quality indicators in Hungary?]. Orv Hetil. 2016;157(9):328–35. Hungarian 10.1556/650.2016.30378 26895800

[24] Kolozsvári LR, Orozco-Beltran D, Rurik I (2014). Do family physicians need more payment for working better? Financial incentives in primary care. Aten Primaria.

[25] Baron RC, Rimer BK, Coates RJ, Kerner J, Mullen PD, Chattopadhyay S, Briss PA (2008). Task force on community preventive services. Methods for conducting systematic reviews of evidence on effectiveness and economic efficiency of interventions to increase screening for breast, cervical, and colorectal cancers. Am J Prev Med.

[26] Spadea T, Bellini S, Kunst A, Stirbu I, Costa G (2010). The impact of interventions to improve attendance in female cancer screening among lower socioeconomic groups: a review. Prev Med.

[27] Bellinger JD, Millegan W, Abdalla AE (2015). “I'm not ashamed to talk on it!”: African-American women's decisions about cervical cancer prevention and control in South Carolina. Womens Health.

[28] Crawford J, Ahmad F, Beaton D, Bierman AS (2016). Cancer screening behaviours among south Asian immigrants in the UK, US and Canada: a scoping study. HSCC.

[29] Arbyn M, Anttila A, Jordan J, Ronco G, Schenck U, Segnan N (2008). European Guidelines for Quality Assurance in Cervical Cancer Screening.

[30] Döbrőssy L, Kovács A, Budai A, Odor A, Fehér E (2013). Screening for cervical cancer in Hungary: new role for health visitors. Clin Nurs Stud.

[31] Fehér E (2012). A Védőnői Méhnyakszűrő Programok 3 éve [3 years of Health Visitor ‘s Cevical Screening Programs]. Védőnő.

[32] Smith JL, Wilson KM, Orians CE, Byrd TL (2013). AMIGAS: building a cervical cancer screening intervention for public health practice. J Womens Health.

