# Supplementary material for: General practitioners can increase participation in cervical cancer screening – a model program in Hungary
Source: BMC Fam Pract. 2018 May 19;19:67. doi: 10.1186/s12875-018-0755-0 (PMC5960501; doi:10.1186/s12875-018-0755-0)
Supplement: Supplementary file 1 — Questionnaire. (DOCX 44 kb) [file 12875_2018_755_MOESM1_ESM.docx]

NPHMOS Code:

Cardboard trunk code sticker place:

QUESTIONNAIRE

Department of Preventive Medicine, Faculty of Public Health, University of Debrecen

*Please read the introductory text carefully, there are important information that will help to fill in the questionnaire!*

*Only your general practitioner could identify you personally by the NPHMOS (National Public Health and Medical Officer's Service) Code and the cardboard number that can be found on the questionnaire.* *However, general practitioners do not have access to the completed questionnaires or to a database which contains the answer.*

*If you are agree to this in the Declaration of Conduct, your general practitioner will be informed by on the basis of your card number, to what extent you use public health cervical cancer screening.*

*When filling in the questionnaire, please circulate the letter of the answers that you find the most appropriate. In the squares, enter the asked quantity and the short, concise answer on the drawn line.*

*Please only indicate one of the answers that you find the most appropriate one for each question! If multiple responses can be identified for a question, this will be indicated by a separate accompanying text.*

*For the sake of appreciation, please answer all the questions if it is possible.*

*Please answer even if you feel that the question is not closely related to the topic of the survey.* *The questions have been drawn up by experts and the questions are closely linked to the subject of the survey.*

*Of course, you can refuse to answer any of the questions in the questionnaire. In this case our colleagues will only evaluate the questions that you answered.*

*If you have accidentally selected a wrong answer while filling in the questionnaire, please let us know the change clearly.*

*Please remember to sign the Declaration in both places and post it together with the questionnaire.*

*In the following, we would like to ask you some questions about your marital status, education and occupation.* *Of course, this information is just like the other response is treated confidentially.*

1. Year of birth: 
2. The ZIP code of your place of residence (where you actually live):
3. Please select from the following those who you live with!

*You can circle more answers in this question.*

1. I am single
2. Married
3. Partner
4. Parent/Parents
5. Child/Children
6. Other relative/ Relatives
7. Others (please write here):
8. What is your highest level of education?
9. Less than primary school
10. Primary school
11. Vocational school
12. Secondary Technical school
13. Secondary Grammar School
14. Professional qualifications
15. Bachelor's Degree/ College degree
16. University degree

*The following question is not only for you but those who you live with. We mean those people who you live in the same flat and live in whole or in part from common income. We will also consider members of the household who are not your relatives but meet the above conditions.*

1. Including you, how many people live in your household?

People

1. Please estimate how much is your total household monthly net income, how much money can they spend on a monthly basis?

HUF

1. Do you work recently? (we mean that is at least one hour a week of earning activity regardless of whether there is a legal relationship with other income, eg retired)
2. Yes *-----------------------------------> Please continue with question 9*
3. No
4. What is the reason why you are not currently working?
5. I'm unemployed, I cannot find a job
6. I get maternity leave, child care allowance, child care benefit
7. I receive retirement pension and/or widow pension
8. I am a disabled pensioner
9. I am a student/ student of higher education
10. I live from other income
11. I get social aid, I am dependent, my family supports
12. I get a nursing fee
13. Other (please write here):
14. What do you do? If you do not have a job now, what your last job was.
15. Intellectuals (Lawyer, Doctor, Engineer, Teacher)
16. Other intellectual (office worker, agent, etc.)
17. Vocational (agricultural)
18. Vocational (not agricultural)
19. Semi-skilled worker (agricultural)
20. Semi-skilled worker ( not agricultural)
21. Unskilled worker
22. A housewife, a student or never done paid work
23. Other (please write here):

*We would like to survey some of your lifestyle features with the following questions.*

*First of all you are asked about if you did any intensive or less intensive sports in the last 7 days. Please answer all the question even if you think you are not so sporty. Think about not only for going for a trip and sports but for all kinds of physical exercises you do connecting with your job, home or round the house as well.*

*First you are asked about INTENSIVE* *physical exercise. During intense physical activity, we mean a movement that requires great effort when breathing is significantly faster than normal, he/she perspires or noticeably accelerating its heartbeat.* *For example: heavy load loading, construction work, heavy garden work, running, fitness sports.*

1. How many days were there in the last 7 days when you did INTENSE exercise at least 10 minutes?

 Day

1. On these days, how much time did you spend with exercise on one or more occasions?

Hours Minute

*The following two questions* refers to MODERATE *physical activity.* *During moderate exercise, we mean movement requiring moderate effort when breathing is somewhat more frequent than normal. For example: cleaning, kitchen garden cultivation, walking, gardening, swimming, stair climbing.*

1. How many days were there in the last 7 days when you did MODERATE exercise at least 10 minutes?

 Day

1. On these days, how much time did you spend with moderate exercise on one or more occasions?

Hours  Minute

1. How tall are you without shoes?

**** cm

1. What is your weight without clothes or shoes?

**** kg

*The following questions are about your smoking habits.*

1. Do you smoke a cigarette, a pipe or cigar?
2. Yes, daily
3. Yes, sometimes in a week -------> *Please continue with question 18th*
4. Less than a week *----------------> Please continue with question 18th*
5. I used to smoke but I gave up *--> Please continue with question 18th*
6. No, I have never smoked -*------> Please continue with question 18th*
7. About how many cigarettes do you smoke a DAY?
8. None, because I do not smoke cigarettes
9. Less than a pack (under 20)
10. About one pack (20)
11. About one and a half pack (30)
12. About 2 packs (40)
13. About 3 packs (60)
14. More than 3 packs (more than 60)

*The following questions are about your sexual life.* *Please, if possible, answer all the questions even if you find them too confidential because it is very important for the test!*

*Again, please note that after posting them, no one can identify in any ways, who has completed the questionnaire.*

1. Have you ever had a sexual relationship with a man?
2. Yes
3. No*-----------------------------------> Please, continue with question 24th*
4. How old were you when you first had sexual relation with a man?

 Age

1. How many men did you have sexual intercourse in the past 12 months?
2. No one
3. One
4. Two
5. Three
6. Four
7. Five
8. More than five
9. Do you currently live with a regular sex life with a man?
10. Yes
11. No*-----------------------------> Please continue with question 24th*
12. Please circle from the below list which contraception methods do you use together with your partner.

*You can circle more than one answers in this question*

1. We do not use any kinds of contraception
2. I regularly take birth control pills
3. Contraceptive medicine which is implanted under the skin
4. Contraceptive injection
5. Contraceptive patch
6. Intrauterine device (for example: spiral, loop)
7. Post-event pill
8. Condom
9. Pessary
10. Vaginal spermicides (foam, cream, jelly, cone)
11. Interrupted intercourse
12. Calendar Method
13. Other (please write here):
14. What kind of diseases have you had from the followings?

*You can circle more than one answers in this question as well*

1. Chlamydia
2. Herpes appearing genitals
3. Verruca appearing genitals
4. Gonorrhea
5. Syphilis
6. Bacterial vaginal infection
7. Candida
8. Trichomonas infection
9. Abnormal vaginal discharge
10. Other (please write here):
11. I have not had any of them

*The following questions are about your general health condition*

1. Do you have any complains, injuries, diseases which hinder or restrict you in your normal activities, such as working, shopping, thing to do, do some sports or keep in touch with others?
2. Have
3. Do not have *-----------------> Please continue with question 26th.*
4. How long does this limitation exist?
5. Up to two weeks
6. More than two weeks, but less than a year
7. More than a year
8. In your opinion, how much can you do for your health?
9. Very much
10. A lot
11. Little
12. Nothing

*Next, here are some questions about the use of some healthcare services*

1. When did you last see your GP to check your health condition but you were not ill?
2. Never
3. More than two years ago
4. One or two years ago
5. Less than a year ago
6. When did you last visit your gynecologist in order to check your health without having any problems?
7. Never
8. More than two years
9. One or two years ago
10. Less than a year ago
11. How old were you when you first visit a gynecologist?

 Age

1. I have never visited a gynecologist *---> Please continue with question 31st*
2. When were you first examined by a gynecologist?
3. Within a year *-----------------------> Please continue with question 32nd*
4. Within three years *------------------> Please continue with question 32nd*
5. More than three years ago
6. What is the reason for not visiting a gynecologist regularly?

*You can circle more than one answers in this question*

1. I do not thing it is important
2. I do not have any problems
3. I do not have time for it
4. There are not any gynecologists near my home
5. I do not have money for it
6. I have bad experiences with gynecologists
7. I only visit a gynecologist if there is a problem
8. Have you been smeared for cytological examination, i.e. screening for cervical cancer?
9. Yes, within a year
10. Yes, within three years
11. Yes, more than three years ago
12. No, never*----------------------------> Please continue with question 38th*
13. Were you examined because of your complaints?
14. Yes
15. No
16. Were you informed about the result of the examination?
17. Yes, I asked it.
18. Yes, I got a notification about it.
19. No
20. Have you already been diagnosed with abnormal cervical cytology?
21. Yes
22. No*---------------------------------> Please continue with question 38th*
23. I was not informed about any results of my examinations*--------> Please continue with question 38th*
    When were you first diagnosed with abnormal cervical cytology?



1. What kind of abnormal cervical cancer cytology have you already had?

*You can circle more than one answers in this case.*

1. ASCUS or ASCH
2. AGCUS or AGCH
3. AIS
4. LSIL
5. HSIL
6. CIN I
7. CIN II
8. CIN III
9. CIN IV
10. CIS
11. P3
12. P4
13. P5
14. I had an abnormal medical record but I do not know what kind it was.
15. Have you ever received a written invitation to cervical cancer screening?
16. Yes
17. No*------------------------------------> Please continue with question 41st*
18. Did you go to the screening test?
19. Yes*-----------------------------------> Please continue with question 41st*
20. I have just got the invitation, I will go*-> Please continue with question 41st*
21. No
22. What is the reason for not taking part in the screening test?

*You can circle more than one answers in this case.*

1. I regularly go to a gynecologist who does the examination
2. I had a kind of gynecological surgery which is why I no longer need to go for a screening test any more
3. I could not find the right time
4. You should have to travel far for testing
5. I did not consider it was important to go
6. I am afraid of the examination
7. I am afraid of the result of the examination
8. I had bad experience about it
9. I have no complaints, so I have no reason to go
10. I have not got enough information about the examination
11. I do not want to take part in any kind of gynecological examinations
12. Other reason (please write here):
13. Please, indicate how much you agree with the following statements.

|  | **I totally disagree** | **I partially agree** | **I totally agree** |
| --- | --- | --- | --- |
| **41_1. Frequent gynecological problems and illnesses can be best prevented by screening** | a | b | c |
| **41_2. At least once a year you have to go to a gynecological screening** | a | b | c |
| **41_3. I find it difficult to understand if someone does not regularly go to** **screening test** | a | b | c |

1. Do you know that every 25-65 years old woman is entitled to cervical cancer screening totally free of charge, without paying once in every three year in public health care in Hungary?
2. I do not know it
3. Yes, I know it
4. If the gynecological smear screening that are needed for cancer screening is done by qualified regional nurses where you live, would you use this service once in every 3 year?
5. Yes
6. I would not take part in the screening, unless the gynecologist takes it
7. I would continue to go to my own gynecologist
8. I would not take part in any gynecological examinations
9. Have you already looked for or asked for any information about cervical cancer screening?
10. Yes
11. No*------------------------------------> Please continue with question 47th*
12. How did you get information about cervical cancer screening?

*You can circle more than one answers in this case.*

1. From health visitor
2. From GP
3. From gynecologist
4. At human papillomavirus ambulance
5. From oncologist
6. From pharmacist
7. From a civil organization
8. From family members
9. From friends, acquaintances, colleagues
10. From leaflets in a doctor's surgery
11. From TV, radio
12. From newspapers
13. From professional books
14. From the internet
15. Other (please write here):
16. What kind of information did you look or ask about the screening?

*You can circle more than one answers in this case.*

1. Where is this screening done?
2. Who does the screening?
3. Can this screening detect any early changes?
4. Does screening needs any special preparations (such as blood sampling)?
5. How does the screening work, what does the doctor do?
6. Is the screening painless?
7. Is screening risky? Can cause any damages?
8. How long does the examination take?
9. When will be the results of the screening available?
10. How and who will inform me about the result?
11. What does it mean if the result of the screening is unfavorable?
12. What will happen if the result of the screening is unfavorable?
13. Why is it necessary to take part in screening?
14. How often is it advisable to go for screening?
15. From what age is it good to go for screening?
16. Who invites people for screening?
17. Other (please write here):
18. Have you ever talked about cervical cancer screening test with your GYNECOLOGIST?
19. Yes, my gynecologist told me every important information about the screening before I had asked him/her any questions.
20. Yes, but only when I asked the doctor.
21. The doctor did not mention it, and I did not even mention it.
22. I do not visit a gynecologist.
23. Have you ever talked about cervical cancer screening test with your GP?
24. Yes, my GP told me every important information about the screening before I had asked him/her any questions.
25. Yes, but only when I asked the doctor.
26. The doctor did not mention it, and I did not even mention it.
27. No, because I do not visit my GP.
28. Have you ever talked about cervical cancer screening test with your HEALTH VISITOR?
29. Yes, the health visitor told me every important information about the screening before I had asked her any questions
30. Yes, but only when I asked the health visitor.
31. The health visitor did not mention it, and I did not even mention it.
32. I do not meet the health visitor.
33. I do not know the health visitor.

*The following questions concern cervical cancer and its prevention*

1. Indicate whether you think that the following factors increase the risk of developing cervical cancer?

|  | **Increase the risk** | **It does not increase the risk** | **I do not know** |
| --- | --- | --- | --- |
| **50_1. older age** | a | b | c |
| **50_2. regular sexual life** | a | b | c |
| **50_3. change sexual partner** | a | b | c |
| **50_4. smoking** | a | b | c |
| **50_5. unprotected sexual life** | a | b | c |
| **50_6. pregnancy, delivery** | a | b | c |
| **50_7. menopause** | a | b | c |
| **50_8. contraception, taking pills** | a | b | c |
| **50_9. inadequate intimate hygiene** | a | b | c |
| **50_10. occurrence in the family** | a | b |  |

1. How you do feel yourself at risk for cervical cancer?
2. Greatly
3. Average
4. Less
5. Not at all
6. I do not care
7. What do you think of the symptoms of cervical cancer are?

*You can circle more than one answers in this case.*

1. Irregular bleeding
2. Vaginal discharge
3. Lower abdominal pain
4. Low back pain
5. Painful sexual intercourse
6. Bleeding between periods
7. There can be no symptoms
8. I do not know
9. How can cervical cancer be cured?

*You can circle more than one answers in this case.*

1. Anti-tumor medication (cytostatic, chemotherapy)
2. Radiotherapy
3. Operation
4. Incurable
5. I do not know
6. How to prevent cervical cancer??

*You can circle more than one answers in this case.*

1. It cannot be prevented
2. Medicine
3. Healthy lifestyle
4. Vaccination
5. Proper hygiene
6. Without sexual life
7. Using condom
8. Taking vitamins regularly
9. Regular screening and early treatment
10. I do not know

Next, we would like to ask you some questions about the human papilloma virus (HPV)

1. Please indicate according to your opinion, which statements are true for the human papilloma virus.

|  | **Yes** | **No** | **I do not know** |
| --- | --- | --- | --- |
| **55_1. It can infect both men and women** | a | b | c |
| **55_2. Vaccination can prevent viral infection** | a | b | c |
| **55_3. Human papillomavirus can cause cervical cancer** | a | b | c |
| **55_4. Condom use reduces the risk but does not provide 100% protection against the virus** | a | b | c |
| **55_5. Easily spreads during sexual intercourse** | a | b | c |

In the following, we would like to ask you a few questions in relation to VACCINATION.

1. Please, check for the following statements, to what extent you agree with them!

|  | **I totally disagree** | **I partly agree** | **I totally agree** |
| --- | --- | --- | --- |
| **56_1. Vaccinations have much more benefit than risks** | a | b | c |
| **56_2. I feel safe with vaccines against serious diseases** | a | b | c |
| **56_3. Not only compulsory vaccinations should be used, but also other recommended vaccinations** | a | b | c |
| **56_4. The vaccinations reduce the risk of developing the particular disease to a minimum** | a | b | c |

1. If there was a safe vaccine that would likely to prevent cervical cancer, would you vaccinate yourself?
2. Yes
3. I had a gynecological surgery that is why I will not have cervical cancer *------------------------------------------------->Please continue with question 60th*
4. No*------------------------------------> Please continue with question 60th*
5. If you had to pay for the vaccination, how much would you be willing to pay for it?
6. Only if the vaccine is free, I would use
7. 1 - 1 000 HUF
8. 1 001 – 5 000 HUF
9. 5 001 – 10 000 HUF
10. 10 001 – 30 000 HUF
11. 30 001 – 50 000 HUF
12. 50 001 – 100 000 HUF
13. If you wish to use the vaccine, with whom would you vaccinate yourself?

*You can circle more than one answers in this case.*

1. My gynecologist
2. My GP
3. If there was a safe vaccine that would likely to prevent cervical cancer, would you vaccinate your daughter or daughters who are under 18?
4. Yes
5. I do not have a daughter under 18 *---->* *Thank you for your cooperation!*
6. My daughter had a gynecological surgery that is why she will not have cervical cancer----------------------------*--------> Thank you for your cooperation!*
7. No*--------------------------------------> Thank you for your cooperation!*
8. If the vaccination was to be paid, how much would you be willing to pay for your daughter's vaccination?
9. Only if the vaccinate is free, I would use it
10. 1 - 1 000 HUF
11. 1 001 – 5 000 HUF
12. 5 001 – 10 000 HUF
13. 10 001 – 30 000 HUF
14. 30 001 – 50 000 HUF
15. 50 001 – 100 000 HUF
16. If you would use the vaccine, with whom would you vaccinate your daughter/daughters? *You can circle more than one answers in this case.*
17. Gynecologist
18. GP
19. The family pediatrician doctor

**Thank you very much for your cooperation!**
